# Elevated Plasma Levels of Drebrin in Glaucoma Patients With Neurodegeneration

**DOI:** 10.3389/fnins.2019.00326

**Published:** 2019-04-03

**Authors:** Yi-Jing Gan, Ai-Wu Fang, Chang Liu, Bai-Jing Liu, Feng-Mei Yang, Ji-Tian Guan, Chun-Lin Lan, Xiao-Dan Dai, Tong Li, Ying Cao, Yun Ran, Xian-Hui Gong, Zi-Bing Jin, Ren-Zhe Cui, Takeshi Iwata, Jia Qu, Fan Lu, Zai-Long Chi

**Affiliations:** ^1^State Key Laboratory of Ophthalmology, Optometry and Visual Science, The Eye Hospital of Wenzhou Medical University, Wenzhou, China; ^2^International Joint Research Center for Regenerative Medicine and Neurogenetics, Wenzhou Medical University, Wenzhou, China; ^3^Department of Ophthalmology, Affiliated Hospital of Yanbian University, Yanji, China; ^4^Division of Molecular and Cellular Biology, National Institute of Sensory Organs, National Hospital Organization Tokyo Medical Center, Tokyo, Japan

**Keywords:** DBN1, glaucoma, axonal degeneration, RGCs, RNFLD

## Abstract

Glaucoma is an optic neuropathy characterized by progressive degeneration of retinal ganglion cells (RGCs). Aberrations in several cytoskeletal proteins, such as tau have been implicated in the pathogenesis of neurodegenerative diseases, could be initiating factors in glaucoma progression and occurring prior to axon degeneration. Developmentally regulated brain protein (Drebrin or DBN1) is an evolutionarily conserved actin-binding protein playing a prominent role in neurons and is implicated in neurodegenerative diseases. However, the relationship between circulating DBN1 levels and RGC degeneration in glaucoma patients remains unclear. In our preliminary study, we detected drebrin protein in the plasma of glaucoma patients using proteomic analysis. Subsequently, we recruited a total of 232 patients including primary angle-closure glaucoma (PACG), primary open-angle glaucoma (POAG) and Posner-Schlossman syndrome (PS) and measured its DBN1 plasma levels. We observed elevated DBN1 plasma levels in patients with primary glaucoma but not in patients with PS compared to nonaxonopathic controls. Interestingly, in contrast to tau plasma levels increased in all groups of patients, elevated drebrin plasma levels correlated with retinal nerve fiber layer defect (RNFLD) in glaucoma patients. To further explore the expression of DBN1 in neurodegeneration, we conducted experiment of optic nerve crush (ONC) models, and observed increased expression of DBN1 in the serum as well as in the retina and then decreased after ONC. This result reinforces the potentiality of circulating DBN1 levels are increased in glaucoma patients with neurodegeneration. Taken together, our findings suggest that circulating DBN1 levels correlated with RNFLD and may reflect the severity of RGCs injury in glaucoma patients. Combining measurement of circulating drebrin and tau levels may be a useful indicator for monitoring progression of neurodegenerative diseases.

## Introduction

Glaucoma comprises a group of optic neuropathies characterized by progressive degeneration of RGCs (Weinreb et al., 2014), resulting in characteristic changes in the ONH and RNFL, and associated VF defects (Venkataraman et al., 2010; Almasieh et al., 2012; Chen et al., 2017; Choi et al., 2017). The biological basis of glaucoma is poorly understood, and the factors contributing to its progression have not been fully characterized (Nickells et al., 2012). Diagnosis of glaucoma is made by a combination of identifying characteristic changes of the RGCs and ONH, functional testing such as VFs, and structural imaging of the retina (Mantravadi and Vadhar, 2015). Sometimes, however, OCT analysis and the VF test are infeasible because of refractive medium opacity and unexpected conditions. More importantly, as many as 30 to 50% of RGCs may be lost before VF defects are detectable in clinical examination (Weinreb et al., 2014). Early diagnosis and treatment of glaucoma are key to preventing vision loss. To date, no biochemistry indexes have been used for diagnosis or evaluation of the severity of axonal injury or axonopathy.

Axonal transport deficits or axonopathy is a component of early glaucoma pathogenesis. Aberrations in several cytoskeletal proteins, such as neurofilaments, microtubules, and tau, have been implicated in the pathogenesis of neurodegenerative diseases, could be initiating factors in glaucoma progression and occurring prior to axon degeneration (Wilson et al., 2016). Previous studies have suggested that tau protein and Aβ may serve as both diagnosis and prognosis biomarkers for the most common neurodegenerative disorder AD (Perrin et al., 2009; Jack et al., 2010; Nucci et al., 2010). Glaucoma progression is associated with altered CSF levels of tau proteins (Chiasseu et al., 2016). Several studies have also revealed links between AD and glaucoma; neuropathological progression occurs in the brain as well as in the eye and is mediated by a common neurodegenerative pathway (Javaid et al., 2016). Tau is a cytosolic protein predominantly expressed in neurons and regulates microtubule stability within the axon. It is increasingly recognized that tau accumulates with age and ocular hypertension glaucoma and displays signature pathological features of tauopathies, leading to neurodegeneration (Chiasseu et al., 2016). However, these pathological hallmarks of neurodegenerative diseases are either measured through CSF analysis with invasive collection or diagnosed postmortem (Javaid et al., 2016).

Drebrin is a cytoplasmic actin-binding protein that plays a role in the process of neuronal growth (Sekino et al., 2007), which is classified into two major isoforms in mammals produced by alternative splicing from a single *DBN1* gene: drebrin A (adult) and drebrin E (embryonic) (Shirao and Sekino, 2017). Drebrin A is neuron specific (Koganezawa et al., 2017) and highly concentrated in dendritic spines (Hayashi et al., 1996), and its accumulation level is regulated by synaptic activity (Takahashi et al., 2009; Takahashi and Naito, 2017). In contrast, drebrin E is found in widespread but not ubiquitous cell types in various tissues (Shirao and Obata, 1986; Ishikawa et al., 1994; Luna et al., 1997; Peitsch et al., 1999). DBN1 decorating filamentous actin (F-actin) is found at the recipient side of cell-cell communication systems, such as neuronal synapses (Shirao and Sekino, 2017). It has been studied that the hippocampal levels of DBN1 in AD mice model were significantly lower than control, implying that DBN1 may be involved in the degeneration of the central nervous system (Liu et al., 2017). Other studies also found that DBN1 levels were significantly decreased in hippocampal synapses and in the frontal and temporal cortex in patients with AD and DS (Harigaya et al., 1996; Shim and Lubec, 2002).

In our preliminary study, we detected DBN1 in the plasma of patients with POAG by proteomic analysis. To investigate the relationship between DBN1 levels and RGC degeneration in glaucoma patients, a correlation analysis has been performed between the DBN1 plasma levels and RNFLD. In this study, we demonstrated that DBN1 plasma levels increased significantly in glaucoma patients with neurodegeneration and were correlated with RNFLD. In contrast, tau plasma levels were elevated in all groups including PS patients with mild RNFLD. Moreover, we also observed elevated DBN1 levels in the serum of ONC model. Our data suggest that DBN1 plasma levels may reflect the severity of RGC damage in glaucoma patients.

## Materials and Methods

### Patients

The study was performed according to the tenets of the World Medical Association’s Declaration of Helsinki and was approved by the Clinical Research Ethics Committee at the Eye Hospital of Wenzhou Medical University. A total of 232 patients including 164 PACG, 46 POAG, and 22 PS patients were recruited at the Eye Hospital of Wenzhou Medical University from October 2016 to December 2017. The nonaxonopathic control group (*n* = 50) comprised patients without any neurodegenerative disease. All participants were questioned about age, gender, history of ocular disease, and systemic clinical symptoms. VF test and IOP were measured bilaterally, and a slit-lamp examination and OCT examination were performed. A consensus was developed before the laboratory and clinical information.

### Inclusion / Exclusion Criteria

(1) All the patients included in this study were diagnosed with PS, PACG, POAG, or cataract (control) by experienced clinical doctors from outpatient.
(1) Only those patients and controls were included who gave their consent for the study either by themselves or with the help of their close kin.
(1) All the subjects under the prescription of other preexisting neurodegenerative diseases either in ocular or nervous system at the time of sample collection were excluded.

### OCT Analysis

**FIGURE 1:**
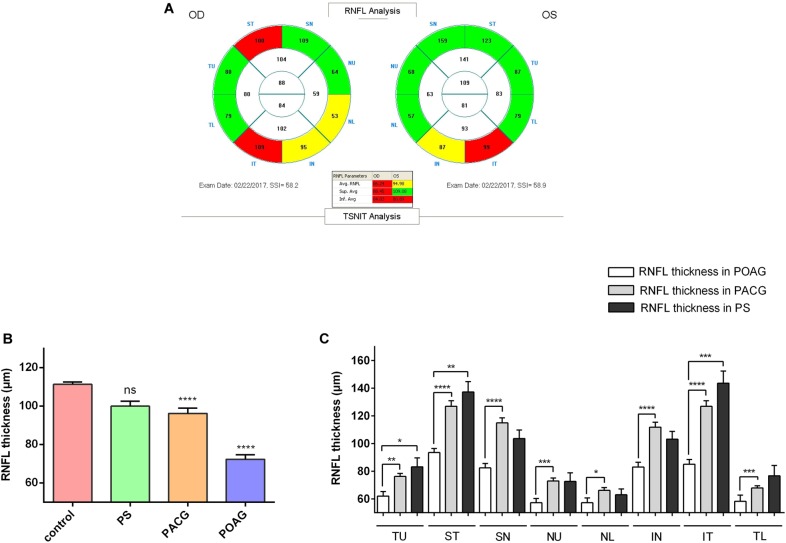
Retinal nerve fiber layer thickness in patients. **(A)** A typical image shown from the OCT scanning result. **(B)** RNFL thickness in control, PS, PACG, and POAG groups. ^∗∗∗∗^*p* < 0.0001 compared to nonaxonopathic control. Mean ± SEM are shown. **(C)** Comparison of the RNFL thickness of the eight quadrants between the glaucoma patients. ^∗^*p* < 0.05, ^∗∗^*p* < 0.01, ^∗∗∗^*p* < 0.001, ^∗∗∗∗^*p* < 0.0001 compared to each other groups. Mean ± SEM are shown.

Optical coherence tomography images were obtained for the RNFL thickness analysis. All OCT images scanning the optic disc were acquired from the RTVue FD-OCT device (Optovue, Inc., Fremont, CA, United States) conducted by experienced operators who were blinded to the research. The RTVue FD-OCT has a light source center wavelength of 830 nm, with a scanning depth of 1.984 mm, maximum scanning width of 6 mm, axial resolution of 5 μm, and scanning speed of 26,000 lines per second. RNFL thickness was divided into eight quadrants TU, ST, SN, NU, NL, IN, IT, and TL (Figure 1A).

### VF Test

All VF tests were performed with a Humphrey 750i automatic field analyzer, with SITA FAST checking strategy. The field analyzer has screen of semispherical shape, with a VF testing distance of 330 mm, cursor of Goldmann III, cursor area of 0.43 mm^2^, background illumination of 31.50 asb, stimulus cursor intensity of 0.008–10,000 asb, and stimulus duration of 200 ms. Results with fixation losses and false-negative and false-positive results ≤ 33% were considered an effective outcome.

### ONC

All animal studies were conducted according to protocols approved by the Institutional Animal Care and Use Committee of Wenzhou Medical University and were in accordance with the ARVO Statement for the Use of Animals in Vision Research. Male Fischer rats and C57BL/6 mice were purchased from Vital River Laboratories (Beijing, China) and were kept in standard cages and fed *ad libitum*. ONC surgery was carried out bilaterally on male 6- to 8-week-old rats (200–230 g) as described (Leon et al., 2000; Yin et al., 2006). The vascular integrity of the retina was evaluated by fundoscopic examination.

### ELISA

The human blood samples were collected in a Vacutainer EDTA tube (BD, Franklin Lakes, NJ, United States). After centrifugation at 2,000 *g* for 10 min at 4°C, the supernatant was transferred and stored at –80°C until analysis. Rat cardiac blood was collected and refrigerated for 2 h, and centrifuged at 2,000 *g* for 10 min at 4°C, the serum was stored in a deep freezer. The protein levels were measured using DBN1 (DL-DBN1-Hu, Dldevelop, Wuxi, China; or KL-DBN1-Ra, Kanglang Biotech, Shanghai, China) according to the manufacturer’s instructions. The minimum detectable dose of DBN1 ELISA kit is 0.047 ng/mL. The standard is recombinant drebrin purified from transformed *E. coli*. The Tau ELISA kit was purchased from Qidibio (Wuhan, China).

### Immunohistochemistry

All the paraffin tissue sections were dewaxed and rehydrated, and blocked with hydrogen peroxide (0.3% H_2_O_2_) for 30 min. Antigen retrieval was performed by heating the slides in sodium citrate buffer for 10 min. Immunohistochemistry analysis were performed using ABC reagent kit (Vector Laboratories, Burlingame, CA, United States). The slides were blocked with prediluted normal horse serum, followed by incubation with anti-drebrin (1:500 dilutions; Abcam, Cambridge, MA, United States) overnight at 4°C. After incubating with a secondary antibody of prediluted biotinylated horse anti-mouse Ig/rabbit Ig for 30 min and R.T.U ABC reagent for another 30 min at RT, DAB was used for color development, and then counterstained with hematoxylin. The stained sections were examined with an inverted microscope (DMi8, Leica, Wetzlar, Germany).

### Statistical Analysis

Characteristics of patients are reported as numbers and percentages for categoric variables and as the mean ± SEM for continuous variables. Experimental data were analyzed using GraphPad Prism or SPSS software. Kruskal–Wallis test was used in age, highest IOP, RNFL thickness and plasma DBN1 and Tau. Chi-square with Bonferroni correction was used for gender comparisons. Logistic regression was used to evaluate the relationship between the DBN1 value and RNFL thickness, which was shown in a meta-analysis (Forest) plot. Statistical differences were analyzed by Student’s *t*-test or Tukey’s *post hoc* test after One-way ANOVA for multiple comparisons of mean values. *p* < 0.05 was considered statistically significant.

## Results

### Characteristics of Patients

General characteristics of glaucoma patients are displayed in Table 1. A total of 232 glaucoma patients and 50 nonaxonopathic controls were recruited for this study. Glaucoma patients as well as controls with preexisting ocular or systemic neurodegenerative diseases were excluded from this study. The mean age of the patients ranged from 44.05 to 64.30 years among the groups. IOP in the glaucoma groups was significantly higher than in the control group. OCT analysis observed that RNFL thickness in POAG and PACG was significantly thinner than in PS patients. The VF test revealed that the VF index (VFI), mean deviation (MD), and pattern standard deviation (PSD) in PS patients was close to normal, whereas a significant loss in PACG and POAG patients was indicated.

**Table 1:** General characteristics of patients.

Variable	Control	PS	*p-*value	PACG	*p-*value	POAG	*p-*value
Number of patients	50	22	/	164	/	46	/
Age (y/o)	60.52 ± 2.14	44.05 ± 2.07	<0.001	64.30 ± 0.77	0.472	50.20 ± 2.25	0.007
Gender (% of female)	52.00	54.55	0.836	75.00	0.002	21.74	0.002
**Highest IOP (mmHg)**							
OD	16.03 ± 1.07	25.37 ± 3.34	0.083	32.10 ± 1.21	<0.001	25.96 ± 1.93	<0.001
OS	14.51 ± 0.68	22.71 ± 2.98	0.084	27.63 ± 1.17	<0.001	23.28 ± 1.70	<0.001
RNFL thickness (um)	111.30 ± 1.18	99.97 ± 2.51	0.245	96.19 ± 2.73	<0.001	72.30 ± 2.36	<0.001
**Visual field test**							
VFI (%)		95.92 ± 1.73	/	63.64 ± 3.28	/	46.68 ± 5.77	/
MD (dB)		-2.91 ± 0.72	/	-13.63 ± 0.95	/	-16.61 ± 2.07	/
PSD (dB)		3.24 ± 0.89	/	5.65 ± 0.29	/	8.07 ± 0.63	/
**Cup to disc ratio**							
OD	0.40 ± 0.06	0.51 ± 0.08	/	0.54 ± 0.02	/	0.77 ± 0.03	/
OS	0.35 ± 0.05	0.43 ± 0.08	/	0.53 ± 0.02	/	0.72 ± 0.03	/


Retinal nerve fiber layer thickness was shown in three colors: within normal was shown in green, borderline was shown in yellow, and outside normal was shown in red, as indicated in OCT reports (Figure 1A). As shown in Figure 1B, among our recruited patients, the RNFLD was severe in POAG and moderate in PACG while close to normal in PS patients. To analyze the characteristics of the RNFL thickness, we divided OCT images into 8 quadrants for analysis. As indicated in Figure 1C, the most significant difference was observed in the ST and IT quadrants in patients with primary glaucoma compared with PS patients.

### Correlation Between DBN1 Levels and RNFL Thickness in Patients With Glaucoma

**FIGURE 2:**
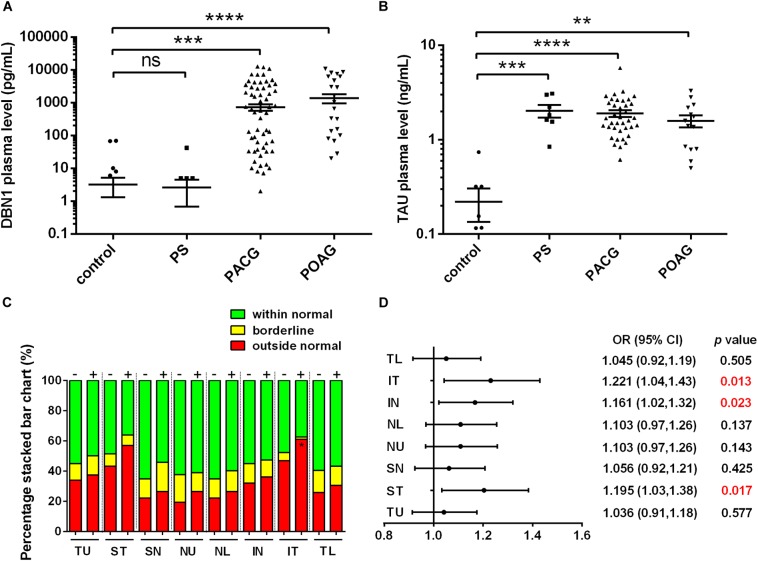
Developmentally regulated brain protein and tau plasma levels in glaucoma patients. **(A)** DBN1 plasma levels in control, PS, PACG, and POAG groups. ^∗∗∗^*p* < 0.001, ^∗∗∗∗^*p* < 0.0001 compared to nonaxonopathic control. Mean ± SEM are shown. **(B)** Tau plasma levels in control, PS, PACG, and POAG groups. ^∗∗^*p* < 0.01, ^∗∗∗^*p* < 0.001, ^∗∗∗∗^*p* < 0.0001 compared to nonaxonopathic control. Mean ± SEM are shown. **(C)** Percentage stacked bar chart shows severity of RNFLD of DBN1-negative (–) and –positive (+) patients. RNFLD in ST quadrant shows significant difference between DBN1-negative (–) and –positive (+) patients (^∗^*p* < 0.05). **(D)** Meta-analysis (forest) plot of logistic regression analysis shows DBN1 plasma levels and RNFL thickness as a dichotomized dependent variable in eight quadrants.

Interestingly, similar to the severity observed during the clinical observation, DBN1 plasma levels were the highest in POAG (1.370 ± 0.412 ng/mL) and increased significantly in PACG (0.721 ± 0.160 ng/mL) patients compared with controls (0.003 ± 0.002 ng/mL) as well as PS (0.003 ± 0.002 ng/mL) (Figure 2A). Meanwhile, for comparison, we also measured the plasma levels of tau protein in those patients (Figure 2B). The mean values of tau proteins increased significantly in all three groups of PACG (1.897 ± 0.155 ng/mL) and POAG (1.582 ± 0.230 ng/mL) as well as in PS (2.022 ± 0.306 ng/mL) patients compared with controls (0.221 ± 0.086 ng/mL). In contrast to DBN1, circulating tau levels increased in PS patients more significantly, and the levels in POAG and PACG were also inconsistent with RNFLD and VF defects.

To further explore the relationship between DBN1 levels and the severity of glaucoma, we divided glaucoma patients into two groups of DBN1-negative and DBN1-positive patients. Based on the detection sensitivity of the kit, patients with DBN1 levels that were under the detectable threshold were regarded as negative. The percentage stacked bar chart showed a more obvious tendency in IT and ST quadrants such that the percentage of outside normal (red) in the DBN1-positive group was higher than in the DBN1-negative group (Figure 2C). To verify the relationship between DBN1 levels and RNFL thickness, we performed logistic regression analyses with RNFL thickness as a dichotomized variable in eight quadrants. Meta-analysis (Forest) plot (Figure 2D) showed a significant difference in the ST, IN, and IT quadrants (*p* < 0.05) with high odd ratio values and 95% confidence intervals.

### Circulating DBN1 Levels Increased in ONC Model

**FIGURE 3:**
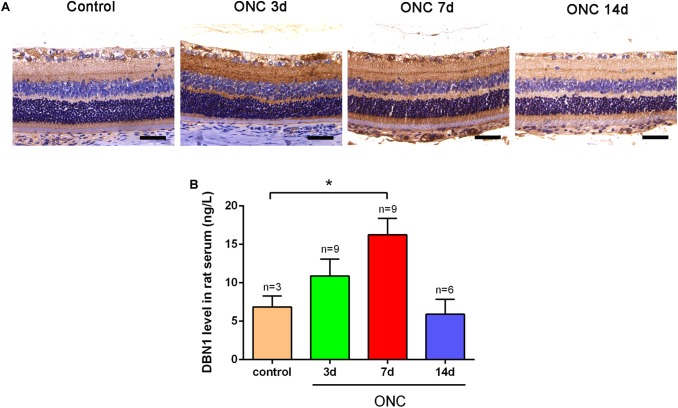
Expression changes of DBN1 proteins in the serum and retina of ONC models. **(A)** Immunohistochemistry of DBN1 in the retina of ONC model. Bars indicate 50 μm. **(B)** Serum DBN1 levels in ONC model. ^∗^*p* < 0.05 compared to control. Mean ± SEM are shown.

To verify the expression of DBN1 proteins after RGC injury, we generated an ONC model, which is frequently used for optical neuropathy studies. Immunohistochemistry showed that DBN1 protein was expressed in whole retina and higher in the RGC layer and IPL, it increased from 3 to 7 days, while decreased at 14 days after ONC (Figure 3A). Similar to expression changes in the retina, DBN1 serum levels increased 3 to 7 days after ONC (*p* < 0.05) as the degeneration of RGCs progressed, and they decreased at 14 days (Figure 3B). These findings suggest that DBN1 protein is released in the circulating blood with neurodegeneration.

## Discussion

Glaucoma represents a group of neurodegenerative diseases characterized by RGC degeneration (Almasieh et al., 2012; Weinreb et al., 2014; Choi et al., 2017). The biological basis of glaucoma is poorly understood, and the factors contributing to its progression have not been fully elucidated. Elevated IOP is considered to be the most important risk factor for glaucoma. One of the known mechanisms of axon degeneration is that nerve insults lead to impaired axonal transport from the cell body, which results in cytoskeletal breakdown, and this process depends on calcium influx and calpain-mediated degradation of DBN1 (Weinreb et al., 2014; Chimura et al., 2015; Choi et al., 2017). Axonal degeneration in glaucoma comprises secondary neurodegenerative events, such as glial activation, homeostatic dysfunction, and neuroinflammation (Tezel and the Fourth ARVO/Pfizer Ophthalmics Research Institute Conference Working Group, 2009; Tezel, 2011, 2013). To date, no biomarkers have been used for diagnosis or evaluation of axonal degeneration of glaucoma. Identification of molecular biomarkers is important to evaluate the severity of RGC axonopathy and may be useful for early diagnosis and prognosis of glaucoma.

Tau plays a pivotal role in axonal transport through regulation and stabilization of microtubule dynamics in neurons, and tauopathy has been extensively studied in the retina (Ho et al., 2012). Recent evidence indicates that altered CSF circulatory dynamics can reduce the clearance of both Aβ and tau, whereas decreased Aβ and increased tau levels in the CSF have been associated with the risk of rapid glaucoma progression (Yang et al., 2010). In this study, we observed that tau plasma levels increased significantly in glaucoma patients compared with nonaxonopathic controls (Figure 1). This is consistent with the observation that tau plasma levels in AD as well as in acute brain injury were significantly elevated compared with normal subjects (Zetterberg et al., 2013). However, the tau plasma level did not precisely reveal the severity of the RGC injury in PS patients, and those levels were also significantly increased in patients with mild RNFLD (Figure 2B).

Our preliminary study detected DBN1 in the plasma of POAG patients using proteome analysis. Subsequently, we recruited over two hundred of glaucoma patients and measured DBN1 plasma levels. In this study, we observed that DBN1 plasma levels increased significantly in glaucoma patients with RNFLD but not in patients without RNFLD or nonaxonopathic controls (Figure 1). To investigate the relationship between DBN1 plasma levels and RGC degeneration in glaucoma patients, we conducted OCT analysis and performed a correlation study. In general, the VF test is important in the evaluation of visual loss in glaucoma patients, but it is sometimes unreliable because of the opacity of refractive media and training differences among patients. Therefore, we used a more objective method based on morphologic, anatomic OCT images. Interestingly, DBN1 plasma levels were positively correlated with RNFLD, especially in IT and ST quadrants (Figure 2).

Drebrin A is specific to neurons and postsynaptic terminals in the adult brain (Harigaya et al., 1996) and highly accumulated within dendritic spines, and regulates actin dynamics (Mizui et al., 2014). DBN1 also plays a role in inhibiting Aβ generation or accelerating its degradation (Liu et al., 2017). It is has been reported that DBN1 mediate the scission of ectosomes from cilia tips (Nager et al., 2017). Neuronal DBN1 levels in the brain are reduced in patients with AD (Harigaya et al., 1996) and DS (Shim and Lubec, 2002), suggesting that DBN1 may serve as an important molecular indicator of brain pathophysiology (Chimura et al., 2015). Numerous laboratory studies as well as human clinical evidence have demonstrated that decreased DBN1 expression in the brain results in cognitive decline (Liu et al., 2015, 2017; Koganezawa et al., 2017; Shirao et al., 2017). Our study also demonstrated that DBN1 was expressed especially in the RGC layer and IPL of retina and its expression was increased at early stages and then finally decreased in ONC model (Figure 3A).

The embryonic-type (drebrin E) is expressed in the embryonic and early postnatal brain and is replaced by the adult-type (drebrin A) during development. Drebrin A is a minor isoform in the early embryonic brain, but the expression increases in parallel with synapse formation. In the adult brain, drebrin A is the major isoform and is highly concentrated in dendritic spines. Although drebrin E is the major isoform in the embryonic brain, some expression continues in the adult brain (Kajita et al., 2017). In this study, our recruited subjects all from adult patients. Although some of the drebrin E may still exist like as in SVZ of brain (Shirao et al., 2017) and may also express in the growing axons after RGC injury. On the other hand, neurons in the adult central nervous system regenerate poorly after damage, suggests that regeneration after RGC injury is limited (Dietmar and Marco, 2012; Norsworthy et al., 2017). Moreover, we cannot be sure that the released drebrin in the blood all from neurons, because that drebrin E is also expressed in non-neuronal cells. However, the drebrin levels are very low in control group as well as PS patients with slight damage in RGCs.

To clarify the correlation of circulating DBN1 levels and RGC degeneration, we subsequently performed an *in vivo* study. The ONC animal model is an experimental disease model for optic neuropathy, which can lead to axonal degeneration, followed by a gradual death of RGCs (Tang et al., 2011). In this study, we observed that DBN1 serum levels increased significantly in ONC rats at days 3 to 7 and then decreased at day 14 after injury (Figure 3B). This result allows us to speculate that circulating DBN1 levels may depend on the severity of RGC degeneration and timing after injury. Thus, our data reveal that DBN1 plasma levels increased with axonopathy of glaucoma patients and then decreased with the progress of axonal degeneration. However, there are also some restrictions, such as correlation with the VF test and follow-up study. Interestingly, previous study have reported that the cytoskeletal proteins, such as tau, neurofilament, calpain-mediated spectrin breakdown product, β-tubulin, and Aβ were changed expressions not only in retina and optic nerve but also throughout the entire retinal projection (primary projection target in mouse, the superior colliculus) in glaucoma mouse model (Wilson et al., 2016). This result gives us some idea of the DBN1 sources in glaucoma patients with neurodegeneration.

In summary, we reported a novel finding that DBN1 plasma levels increased in glaucoma patients. Moreover, DBN1 plasma levels correlated with RNFLD in glaucoma patients and may reflect the severity of RGC injury. DBN1 plasma levels may relate to the timing after injury and RGC damage progression. In conclusion, combining the measurement of circulating DBN1 and tau levels may be a useful indicator for the diagnosis or evaluation of the severity of axonopathy in neurodegenerative diseases.

## Data Availability

All datasets generated for this study are included in the manuscript and/or the supplementary files.

## Ethics Statement

The study was performed according to the tenets of the World Medical Association’s Declaration of Helsinki and was approved by the Clinical Research Ethics Committee at the Eye Hospital of Wenzhou Medical University. All animal studies were conducted according to protocols approved by the Institutional Animal Care and Use Committee of Wenzhou Medical University and were in accordance with the ARVO Statement for the Use of Animals in Vision Research.

## Author Contributions

Z-LC and Y-JG designed the experiments. A-WF, X-HG, R-ZC, B-JL, F-MY, J-TG, C-LL, YC, and YR recruited the patients and collected the blood samples. Y-JG, CL, X-DD, TL, Z-BJ, and TI carried out experiments and acquired and analyzed the data. Z-LC, FL, and JQ designed the study. Z-LC and Y-JG drafted the manuscript.

## Conflict of Interest Statement

The authors declare that the research was conducted in the absence of any commercial or financial relationships that could be construed as a potential conflict of interest.

## Abbreviations

Aβ: amyloid beta
AD: Alzheimer’s disease
ANOVA: analysis of variance
CSF: cerebrospinal fluid
DAPI: 4’, 6-diamidino-2-phenylindole
Drebrin or DBN1: developmentally regulated brain protein
DS: Down syndrome
ELISA: enzyme-linked immunosorbent assay
HP: high pressure
IN: inferior nasal
IOP: intraocular pressure
IPL: inner plexiform layer
IT: inferior temporal
Neuro-2a: neuroblastoma cells
NL: nasal lower
NU: nasal upper
OCT: optical coherence tomography
ONC: optic nerve crush
ONH: optic nerve head
PACG: primary angle-closure glaucoma
PBS: phosphate-buffered saline
PFA: paraformaldehyde
POAG: primary open-angle glaucoma
RGCs: retinal ganglion cells
RNFL: retinal nerve fiber layer
RT: room temperature
SDS-PAGE: sodium dodecyl sulfate-polyacrylamide gel electrophoresis
SEM: standard error of mean
SN: superior nasal
ST: superior temporal
TBST: Tris buffer saline containing 0.1% Tween-20
TL: temporal lower
TU: temporal upper
VF: visual field
